# Axin-binding domain of glycogen synthase kinase 3β facilitates functional interactions with voltage-gated Na+ channel Na_v_1.6

**DOI:** 10.1016/j.jbc.2025.108162

**Published:** 2025-01-08

**Authors:** Timothy John Baumgartner, Nolan Michael Dvorak, Nana Aboadwe Goode, Zahra Haghighijoo, Mate Marosi, Jully Singh, Aditya Kumar Singh, Fernanda Laezza

**Affiliations:** Department of Pharmacology and Toxicology, University of Texas Medical Branch, Galveston, Texas, USA

**Keywords:** voltage-gated sodium channel, Gsk3β, excitability, electrophysiology, protein–protein interaction

## Abstract

Voltage-gated Na+ (Na_v_) channels are the primary determinants of the action potential in excitable cells. Na_v_ channels rely on a wide and diverse array of intracellular protein–protein interactions (PPIs) to achieve their full function. Glycogen synthase kinase 3β (GSK3β) has been previously identified as a modulator of Na_v_1.6-encoded currents and neuronal excitability through PPI formation with Na_v_1.6 and phosphorylation of its C-terminal domain (CTD). Here, we hypothesized that GSK3β functions as a scaffold in a regulatory PPI complex with the Na_v_1.6 CTD. Mutagenesis screening using the split-luciferase complementation assay indicated that the axin-binding domain (ABD) of GSK3β (262–299) is necessary for complex formation between the Na_v_1.6 CTD and GSK3β, and that residues within this domain are drivers of GSK3β-mediated regulation of the channel. Overexpression of an ABD-GFP fusion construct in human embryonic kidney 293 cells stably expressing Na_v_1.6 significantly reduced Na_v_1.6 nanocluster density compared with GFP alone. In addition, overexpression of the ABD-GFP fusion construct ablates GSK3β-mediated potentiation of Na_v_1.6-encoded currents and alters channel kinetics. Finally, *in vivo* AAV-mediated overexpression of the ABD-GFP construct in the CA1 hippocampal region induced a reduction in maximal action potential firing and an increase in action potential current threshold in a manner resembling previously reported effects of GSK3β silencing in neurons. Taken together, these results not only suggest that GSK3β-mediated regulation of Na_v_1.6 extends beyond transient phosphorylation but also implicates the ABD as a critical regulatory domain that facilitates GSK3β′s functional effects on Na_v_1.6 and neuronal excitability.

Voltage-gated Na+ (Na_v_) channels are the principal regulators of action potential initiation and propagation in excitable cells (1, 2). There are nine isoforms of the Na_v_ channel α-subunit that have currently been described (Na_v_1.1–Na_v_1.9) (3), each filling functionally distinct biological roles based upon tissue expression and subcellular localization. Structurally, the pore-forming α-subunit of Na_v_ channels is composed of four transmembrane domains (DI–DIV), each containing six segments (S1–S6) (1, 4). The nine Na_v_ channel isoforms share a high degree of overall sequence homology in this α-subunit, which plays a central role in governing channel activity. However, the full scope of Na_v_ channel physiological function is achieved through protein–protein interactions (PPIs) between the Na_v_ channel α-subunit and auxiliary proteins (5, 6, 7, 8, 9, 10, 11).

Na_v_ channel binding partners represent a structurally and functionally diverse assortment of proteins including intracellular fibroblast growth factors (7, 12, 13), various kinases (10, 11, 14, 15, 16), and calmodulin (9, 17). These PPIs facilitate various Na_v_ channel biological processes, such as trafficking (11, 18), subcellular compartmentalization (19, 20), and degradation (21). In addition to these biological processes, Na_v_ channel auxiliary proteins confer precise modulation of channel kinetics, significantly contributing to regulation of excitability and repetitive firing. Regulation of Na_v_ channel kinetics is observed to occur through both stable interactions with auxiliary proteins and post-translational modifications (14, 16, 17, 21, 22). Notably, these interactions induce functionally divergent effects among Na_v_ channel isoforms.

Among these auxiliary proteins, glycogen synthase kinase 3β (GSK3β) is a highly conserved serine–threonine kinase that displays abundant expression in the central nervous system (CNS) (23, 24). In addition to its canonical metabolic functions, GSK3β has emerged as a key mediator of neurobiological processes, including neurodevelopment (25), axonal transport (26), and synaptic plasticity (10). Accordingly, dysfunction of GSK3β has been implicated in various neuronal disorders, such as bipolar disorder, schizophrenia, Parkinson’s disease, and Alzheimer’s disease (24, 27, 28, 29, 30).

While full scope of GSK3β′s neurobiological functions is yet to be fully characterized, its effects on some neuronal processes have been attributed to regulation of ion channels. Evidence suggests that GSK3β directly phosphorylates the intracellular loop-connecting domains II and III of hippocampal P/Q-type Ca^2+^ channels, decreasing Ca^2+^ influx and thereby blocking synaptic vesicle release (31). Voltage-gated potassium channel K_v_7.2 has been also identified as a direct substrate of GSK3β, and resultant inhibition of K_v_7.2 has been proposed as a mechanism underlying circuit dysfunction in schizophrenia (32, 33).

Previous investigations from our research group have identified GSK3β as a regulator of CNS Na_v_ isoforms 1.2 (11) and 1.6 (10). Notably, although evidence suggests that GSK3β targets similar regions of both the Na_v_1.6 and Na_v_1.2 channel, the resultant functional effects diverge among the two isoforms. In regard to Na_v_1.2, it is observed that pharmacological inhibition or genetic silencing of GSK3β results in potentiation of Na_v_1.2 currents, whereas GSK3β overexpression results in suppression (11). Conversely, in terms of Na_v_1.6, genetic manipulation or pharmacological inhibition of GSK3β result in suppressed channel activity, whereas GSK3β overexpression potentiates Na_v_1.6-encoded currents (10).

It has been shown that Na_v_ channel auxiliary proteins can elicit functionally diverse isoform-specific effects on channel activity through interactions at their intracellular C-terminal domain (CTD); a region of Na_v_ channels that displays appreciable sequence divergence in the otherwise highly conserved pore-forming α-subunit (34). In addition to GSK3β-mediated phosphorylation of Na_v_1.6 (T1936), our previous studies using biophysical *in vitro* assays suggest that GSK3β binds directly to the Na_v_1.6 CTD.

Building on these initial studies, in the present investigation, we sought to further characterize the GSK3β–Na_v_1.6 PPI complex and identify critical domains of GSK3β that confer functional interactions with the Na_v_1.6 channel. Using mutagenesis screening and split-luciferase complementation, we identified the GSK3β axin-binding domain (ABD) as a critical region for binding with Na_v_1.6. Through a combination of confocal imaging and whole-cell patch-clamp electrophysiology, we characterized the contribution of the ABD to GSK3β′s regulatory effects on Na_v_1.6 channel activity. Finally, we show that *in vivo* AAV-mediated overexpression of the ABD suppresses excitability of CA1 pyramidal neurons, recapitulating the previously reported effects of GSK3β silencing and suggesting that overexpressed ABD acts as a decoy binding to Na_v_1.6 CTD and preventing GSK3β–Na_v_1.6 complex formation.

## Results

### Mutagenesis screen

**Figure 1:**
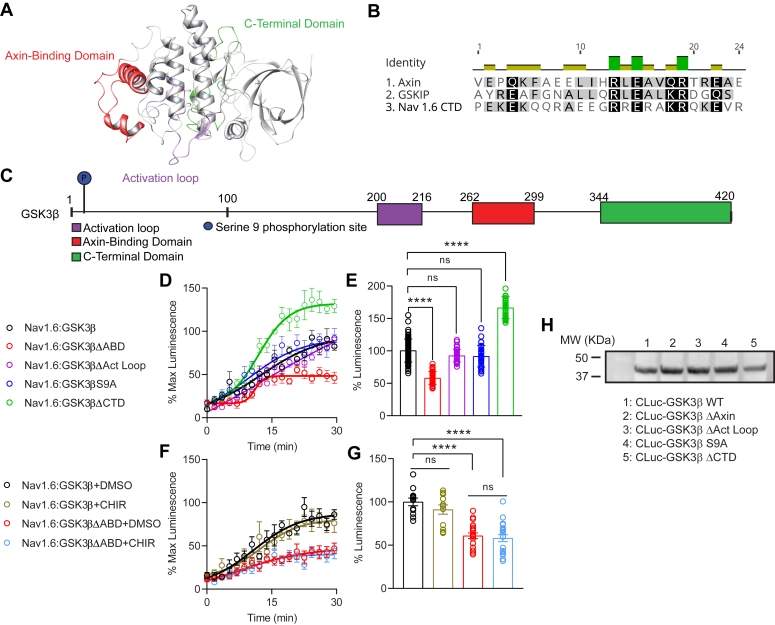
**Mutagenesis screening reveals ABD as a critical motif for GSK3β–Na**_**v**_**1.6 complex assembly.***A*, three-dimensional protein structure of GSK3β. Structural location representation of the GSK3β domains using X-ray crystallography (Protein Data Bank code: 1J1C), illustrating the surface locations of deletion aa 262 to 299 (*red*), activation loop aa 200 to 226 (*purple*), and CTD aa 344 to 388 (*green*). *B*, sequence alignment of the Na_v_1.6 CTD (1952–1976) with GSK3β-binding proteins axin (406–483) and GSKIP (117–139). *C*, schematic of GSK3β sequence indicating position of truncation mutants. *D*, real-time LCA output of GSK3β–Na_v_1.6 complex formation in HEK293 cells transfected with LCA constructs CLuc-GSK3β and CD4-Na_v_1.6-NLuc; d-luciferin was added at time 0. *E*, LCA results of Na_v_1.6 and GSK3β mutant LCA pairs (normalized to controls) reveal a key role of the ABD in mediating GSK3β–Na_v_1.6 complex formation. *F*, real-time LCA output of CD4-Na_v_1.6 Nluc and CLuc-GSK3β or CLuc-GSK3βΔABD treated with vehicle (0.5% DMSO) or 2 μM CHIR99021 (CHIR). *G*, bar-graph representation of the LCA conducted in *F*. *H*, Western blot illustrating expression of GSK3β mutant constructs. Data are mean ± SEM. In *E*; n = 32 individual wells per condition; data are normalized to control (Na_v_1.6:GSK3β, *black*) (Na_v_1.6:GSK3βΔABD, *p* < 0.0001; Na_v_1.6:GSK3βΔAct Loop, *p* = 0.1312; Na_v_1.6:GSK3βS9A, *p* = 0.0636; Na_v_1.6:GSK3βΔCTD; *p* < 0.0001). In *G*; n = 12 to 20 individual wells per condition; data are normalized to control (Na_v_1.6:GSK3β + DMSO, *black*) (Na_v_1.6:GSK3β+CHIR, *p* = 0.5689; Na_v_1.6:GSK3βΔABD+DMSO, *p* < 0.0001; Na_v_1.6:GSK3βΔABD+CHIR; *p* < 0.0001) comparing Na_v_1.6:GSK3βΔABD+DMSO *versus* Na_v_1.6:GSK3βΔABD+CHIR; *p* = 0.9709. Data are represented as mean ± SEM. ∗*p* < 0.05; ∗∗∗*p* < 0.001; ∗∗∗∗*p* < 0.0001 (ordinary one-way ANOVA with *post hoc* Dunnett’s multiple comparisons test). ABD, axin-binding domain; CTD, C-terminal domain; DMSO, dimethyl sulfoxide; GSK3β, glycogen synthase kinase 3β; GSKIP, GSK3 Interacting Protein; HEK293, human embryonic kidney 293 cell line; LCA, luciferase complementation assay.

In the present investigation, we sought to identify critical domains of GSK3β that confer the GSK3β–Na_v_1.6 PPI. To do so, a series of structurally guided truncation were generated (Fig. 1) to identify critical domains for GSK3β–Na_v_1.6 complex assembly. The 3D structure of GSK3β (Fig. 1*A*) is well characterized (35), and there are extensive studies investigating GSK3β′s functional motifs and regions critical for its stable interactions with other proteins (36, 37). Considering this evidence, a series of GSK3β mutants encompassing key functional or binding regions and residues were generated to evaluate their impact on Na_v_1.6 binding (Fig. 1*C*). In previous investigations, we have employed an in-cell luciferase complementation assay (LCA) to assess the formation of Na_v_ channel complexes involving multiple Na_v_ isoforms and auxiliary proteins (38, 39, 40). In the present study, we used the LCA to reconstitute the Na_v_1.6 CTD and GSK3β complex. To do so, human embryonic kidney 293 (HEK293) cells were transiently cotransfected with CD4-Na_v_1.6-CTD-NLuc, a construct that was previously used to reconstitute PPIs in cells (8, 41, 42), and CLuc-GSK3β or one of the GSK3β mutant constructs. The goal of the experiment was to first reconstitute the GSK3β–Na_v_1.6 PPI complex in a cellular milieu, expanding on previous studies showing that the interaction occurred *in vitro* (10) and second to determine the impact of selected mutants on GSK3β–Na_v_1.6 complex assembly.

Our results indicate no significant alterations to GSK3β–Na_v_1.6 complex assembly resulting from alanine mutation of GSK3β serine 9 or deletion of the GSK3β activation loop (Fig. 1, *D* and *E*). These regions are known targets of the AKT-dependent phosphorylation, which inhibits the kinase (43), and are crucial for mediating the enzymatic activity of the kinase (44), respectively. On the other hand, it was observed that deletion of GSK3β′s C-terminal region significantly increased complex formation (166.6 ± 3.0% Lum, n = 32; *p* = <0.0001) with the Na_v_1.6 CTD (Fig. 1, *C* and *E*). This suggests that removal of this region may induce a more favorable conformation for binding to Na_v_1.6 or that may mediate intramolecular interactions within the kinase, unmasking critical binding sites to Na_v_1.6.

Notably, though, deletion of GSK3β′s ABD substantially impairs (42 ± 1.7% Lum, n = 32; *p* = <0.0001) GSK3β–Na_v_1.6 CTD complex formation (Fig. 1, *D* and *E*). Previous biophysical studies describe this region of GSK3β as a conformationally plastic loop that facilitates interactions of the kinase with other proteins and attains divergent stable conformations with each binding partner (45, 46). Moreover, sequence analysis revealed homology between the distal Na_v_1.6 CTD (AA1952–1976) and two other proteins that bind the GSK3β ABD: axin and GSK3 Interacting Protein (GSKIP) (36, 45, 47). Critically, the GSK3β-binding regions of axin and GSKIP share a common RxExxQ/KRxxE/Q motif with the distal CTD of Na_v_1.6 (Fig. S1). Structural analyses of axin and GSKIP in complex with GSK3β indicate that they share common binding sites on GSK3β (45). Notably, Arg395 and Gln400 of axin (corresponding to R1965 and K1970 of the Na_v_1.6 CTD) (Fig. S1) form hydrogen bonds and ionic interactions with D264 and Q295 of GSK3β. Therefore, we hypothesized that the distal CTD of Na_v_1.6 binds to GSK3β *via* a similar mechanism, with orientation of the Na_v_1.6 CTD of Na_v_1.6 into GSK3β′s ABD being conferred by these or surrounding polar residues. Expression of the GSK3β mutant constructs generated was confirmed using Western blot (Fig. 1*H*).

In a previous investigation, we determined that GSK3β phosphorylates T1936 of the Na_v_1.6 CTD (10). Having shown that deletion of the ABD perturbs GSK3β–Na_v_1.6 complex assembly, we next sought to determine the effects of GSK3β inhibition on Na_v_1.6 complex assembly; and if these effects are functionally distinct from those elicited by ABD deletion. To do so, HEK293 cells transfected with CD4-Na_v_1.6-Nluc and either WT CLuc-GSK3β or Cluc-GSK3βΔABD were treated with either vehicle (0.5% dimethyl sulfoxide [DMSO]) or 2 μM CHIR99021 (CHIR) for 1 h prior to evaluation of complex assembly (Fig. 1, *F* and *G*). We observed no significant reduction in GSK3β–Na_v_1.6 complex assembly resulting from treatment with CHIR (91.2 ± 5.4% Lum, n = 12) compared with vehicle control (100.0 ± 4.4% Lum, n = 12, *p* = 0.5689), suggesting that formation of the GSK3β–Na_v_1.6 complex can occur independently of GSK3β enzymatic activity. Deletion of the ABD significantly decreased complex assembly in the presence of either vehicle (60.9 ± 3.3% Lum, n = 20; *p* = <0.0001) or 2 μM CHIR (58.2 ± 4.0% Lum, n = 20; *p* = <0.0001) compared with WT GSK3β; although these groups did not significantly differ from one another (*p* = 0.9532). Taken together, these results indicate that phosphorylation of the Na_v_1.6 CTD is not a requirement for GSK3β–Na_v_1.6 complex assembly, and that the interactions mediated by residues of the ABD occur independently of GSK3β kinase activity.

### Interactions of GSK3**β** ABD mediate Na_v_1.6 clustering

**Figure 2:**
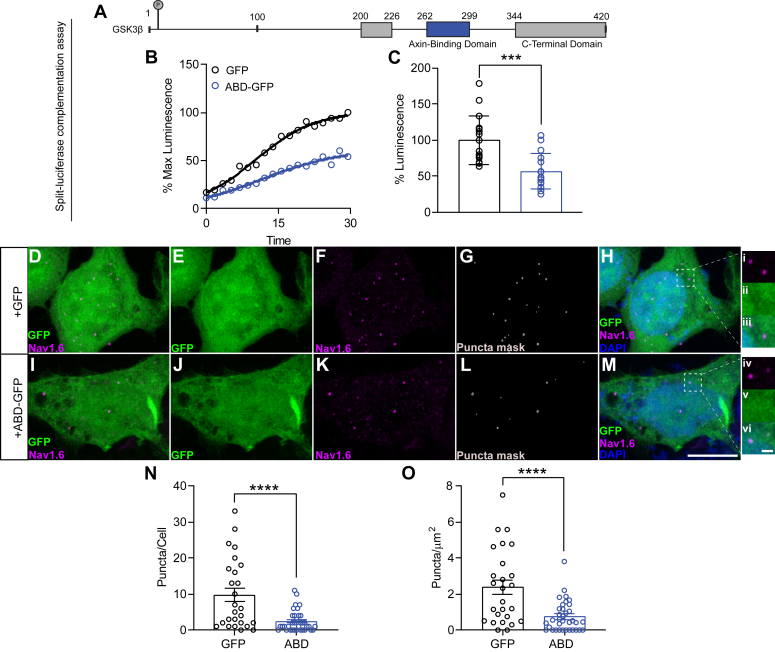
**ABD overexpression decreases GSK3β–Na**_**v**_**1.6 complex assembly and alters Na**_**v**_**1.6 distribution patterns.***A*, schematic of GSK3β sequence indicating the location of the ABD construct. *B*, real-time LCA output of GSK3β–Na_v_1.6 complex assembly in cells overexpressing GFP (*black*) or ABD-GFP (*blue*). *C*, column representation of the LCA in (*B*); each point represents an individual replicate (n = 16 for each condition) (*D*–*I*) confocal images of HEK-Na_v_1.6 cells stably expressing Na_v_1.6 that were transiently transfected with either control (GFP, *D*–*H*) or ABD-GFP (*I*–*M*). Na_v_1.6 puncta were visualized with an Alexa-647-conjugated anti-rabbit Na_v_1.6 antibody. *D*–*F*, show overlays of GFP and Na_v_1.6 (*D*), GFP (*E*), Na_v_1.6 (*F*), puncta masks (*G*), and merged images (*H*) in HEK-Na_v_1.6 cells transiently transfected with GFP control. Panels i–iii indicate *insets* of *F* (i), *E* (ii), and *H* (iii). *I*–*M*, show overlays of GFP and Na_v_1.6 (*I*), GFP (*J*), Na_v_1.6 (*K*), puncta masks (*L*), and merged images (*M*) in HEK-Na_v_1.6 cells expressing ABD-GFP. Panels iv–vi indicate *insets* of *K* (iv), *J* (v), and *M* (vi). *N*, quantification of total puncta/cell in HEK-Na_v_1.6 cells expressing GFP (*black*) or ABD-GFP (*blue*). *O*, quantification of total puncta per/unit area (μm^2^) in cells expressing GFP (*black*) or ABD-GFP (*blue*). In (*N* and *O*), each point represents an individual cell (GFP, n = 35; ABD-GFP; n = 26). Data are represented as mean ± SEM. Significance was assessed using an unpaired *t* test. ∗*p* < 0.05; ∗∗∗*p* < 0.001; ∗∗∗∗*p* < 0.0001. Scale bars represent 10 μm (*M*) and 1 μm (vi). ABD, axin-binding domain; GSK3β, glycogen synthase kinase 3β; HEK, human embryonic kidney cell line; LCA, luciferase complementation assay.

Having shown that deletion of GSK3β′s ABD is sufficient to perturb GSK3β–Na_v_1.6 complex assembly, we hypothesized that overexpression of this region (GSK3β 262–299) (Fig. 2*A*) should occupy the GSK3β-binding region of Na_v_1.6, thereby similarly inhibiting GSK3β–Na_v_1.6 complex assembly. To test this hypothesis, we generated a plasmid coding for the GSK3β ABD (262–299) fused to GFP. Then, HEK cells were transiently transfected with CLuc-GSK3β and CD4-Na_v_1.6-NLuc along with either GSK3β′s ABD (ABD-GFP) or control (GFP) in order to determine the effects of ABD overexpression on GSK3β–Na_v_1.6 complex assembly. Validation of expression for the aforementioned constructs is available in Fig. S1. In agreement with our previous results, overexpression of GSK3β′s ABD resulted in perturbation of GSK3β–Na_v_1.6 complex assembly (Fig. 2, *B* and *C*; 56.45 ± 6.1% Lum; *p* < 0.01); suggesting that the overexpressed ABD may occupy the typical GSK3β-binding region of Na_v_1.6.

Na_v_ channel macromolecular complexes, formed by stable or transient PPIs, are critical in regulating Na_v_ channel subcellular trafficking and compartmentalization (8, 48). In addition, experimental evidence indicates that the Na_v_1.6 channel forms subcellular clusters in cultured neurons, which denote regulated intracellular mechanisms for maintaining channel expression (49). These clusters are important in regulating the surface pool and cellular trafficking to the plasma membrane (49). Previous studies show that GSK3 regulates Na_v_ channel trafficking and cell surface expression (11). Therefore, given that overexpression of the GSK3β ABD perturbs GSK3β–Na_v_1.6 complex assembly, we hypothesized that the subcellular distribution pattern of Na_v_1.6 channels may also be impacted.

To evaluate the effects of GSK3β ABD overexpression on Na_v_1.6 subcellular distribution, we expressed constructs carrying either GFP or the ABD fused to GFP (ABD-GFP) in HEK293 cells stably expressing the Na_v_1.6 channel (HEK-Na_v_1.6 cell line). Following transient transfection of the aforementioned constructs, cells were plated at low density on glass coverslips. Then, Na_v_1.6 channels were labeled using primary Na_v_1.6 antibody followed by a secondary Alexa Fluor 647-conjugated antibody. Then, laser-scanning confocal images were collected from HEK293-Na_v_1.6 cells overexpressing GFP (Fig. 2, *D*–*H*) or ABD-GFP (Fig. 2, *I*–*M*). Investigations of Na_v_ channel distribution in COS7 cells and neurons describe nanoclusters that appear as puncta of ∼1 to 2 μm visualized *via* confocal imaging (48, 49). To observe the effect of ABD overexpression on Na_v_1.6 distribution, cells abundantly expressing GFP were selected and the number of Na_v_1.6 puncta in optical slices encompassing the cell membrane were quantified for each experimental group.

It was observed that HEK-Na_v_1.6 cells overexpressing the ABD-GFP construct contained significantly fewer Na_v_1.6 puncta (Fig. 2*N*; 2.4 ± 0.5, n = 35) than cells expressing GFP alone (9.731 ± 1.9, n = 26; *p* = <0.0001). In addition, to account for any variance in cell size, the number of puncta per unit area was calculated. We observed a similar decrease in the density of Na_v_1.6 puncta distribution per total cell area in cells overexpressing ABD-GFP (Fig. 2*O*; 0.74 ± 0.14 puncta/100 μm^2^, n = 35) compared with GFP alone (2.36 ± 0.40 puncta/100 μm^2^, n = 26; *p* = <0.0001). Intensity plots of Na_v_1.6 immunofluorescence in cells transfected with GFP or ABD-GFP can be found in Fig. S1.

**Figure 3:**
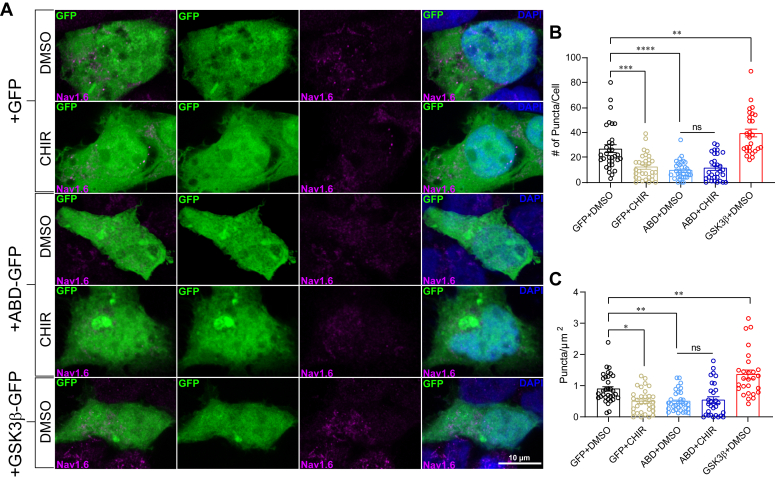
**Effects of GSK3β inhibition and overexpression on Na**_**v**_**1.6 puncta distribution.***A*, laser-scanning confocal images of HEK293 cells overexpressing GFP, ABD-GFP, or GSK3β-IRES-GFP (*green*) and treated with vehicle (0.5% DMSO) or 2 μM CHIR99021 (CHIR). DNA was visualized using 4′,6-diamidino-2-phenylindole (*blue*). Na_v_1.6 puncta were visualized using an Alexa-647-conjugated anti-rabbit Na_v_1.6 antibody (*red*). *B*, quantification of total puncta/cell in the indicated experimental groups. *C*, quantification of total puncta per unit/area (μm^2^) in the indicated experimental groups. Data are represented as mean ± SEM. Significance was assessed using an ordinary one-way ANOVA with multiple comparisons test. In (*B* and *C*), each point represents an individual cell. (GFP+DMSO, n = 33; GFP+CHIR, n = 32; ABD+DMSO, n = 33; ABD+CHIR, n = 30; GSK3β+DMSO, n = 27). In (*B*), GFP-DMSO *versus* GFP+CHIR, *p* = 0.0002; GFP+DMSO *versus* ABD+DMSO, *p* < 0.0001; GFP+DMSO *versus* GSK3β+DMSO, *p* = 0.0025; ABD-DMSO *versus* ABD+CHIR, *p* = 0.9709). In (*C*), GFP-DMSO *versus* GFP+CHIR, *p* = 0.0109; GFP+DMSO *versus* ABD+DMSO, *p* = 0.0090; GFP+DMSO *versus* GSK3β+DMSO, *p* = 0.0054; ABD-DMSO *versus* ABD+CHIR, *p* = 0.9944. ∗*p* < 0.05; ∗∗*p* < 0.01; ∗∗∗*p* < 0.001; ∗∗∗∗*p* < 0.0001. ABD, axin-binding domain; DMSO, dimethyl sulfoxide; GSK3β, glycogen synthase kinase 3β; HEK293, human embryonic kidney 293 cell line.

The effects of ABD overexpression on GSK3β–Na_v_1.6 complex assembly suggest that the alterations to Na_v_1.6 puncta distribution are resultant of perturbing interactions between Na_v_1.6 and natively expressed GSK3β. Thus, we next sought to determine the effects of GSK3β inhibition on Na_v_1.6 puncta distribution (Fig. 3). To do so, laser-scanning confocal images were acquired from HEK293 cells expressing GFP or ABD-GFP and treated with either vehicle (0.5% DMSO) or 2 μM CHIR, and puncta were quantified using the same protocol as in the previous experiment (Fig. 3*A*). We observed fewer Na_v_1.6 puncta following treatment with CHIR (12.3 ± 1.8, n = 32) to vehicle control (26.8 ± 3.1, n = 33; *p* = 0.0002) along with decreased density distribution in cells expressing GFP alone (Fig. 3, *B* and *C*). In addition, in agreement with the previous experiment, fewer Na_v_1.6 puncta were observed in cells overexpressing the ABD (9.5 ± 1.3, n = 33) in the presence of vehicle. Notably, however, although treatment with CHIR decreased Na_v_1.6 puncta in GFP cells, there was no further decrease in Na_v_1.6 puncta following treatment with CHIR in cells overexpressing the ABD (11.5 ± 1.9, n = 30; *p* = 0.9709).

Finally, having observed that inhibition of GSK3β perturbs Na_v_1.6 puncta distribution, we hypothesized that GSK3β overexpression would elicit an opposing effect. To test this, an additional set of images were acquired from HEK-Na_v_1.6 cells transiently transfected with GSK3β-IRES-GFP and treated with vehicle (0.5% DMSO) (Fig. 3*A*). We observed a significant increase in puncta number (Fig. 3*B*) (39.6 ± 3.4, n = 27, *p* = 0.0025) and density distribution (Fig. 3*C*) (1.4 ± 0.14 puncta/μm^2^, n = 27; *p* = 0.0054) in cells overexpressing GSK3β compared with GFP-DMSO control.

Taken together, these observations suggest that GSK3β is a critical facilitator of Na_v_1.6 clustering and cell surface trafficking, and that ABD overexpression inhibits these regulatory functions through perturbation of GSK3β′s interactions with the Na_v_1.6 CTD.

### Functional modulation of Na_v_1.6 by GSK3**β** ABD

Previous studies from our group show that GSK3β is a positive regulator of Na_v_1.6-encoded currents and that GSK3β knockdown results in opposing phenotypes (10). Having shown that overexpression of the GSK3β ABD perturbs GSK3β–Na_v_1.6 complex assembly, we hypothesized that ABD overexpression would also prevent GSK3β-mediated functional potentiation of Na_v_1.6 currents. To investigate the effects of ABD overexpression on Na_v_1.6 activity, HEK-Na_v_1.6 cells were transiently transfected with either ABD-GFP or GFP alone. Then, whole-cell patch-clamp electrophysiology was employed to assess the effects on Na_v_1.6-encoded currents and channel kinetics.

**Figure 4:**
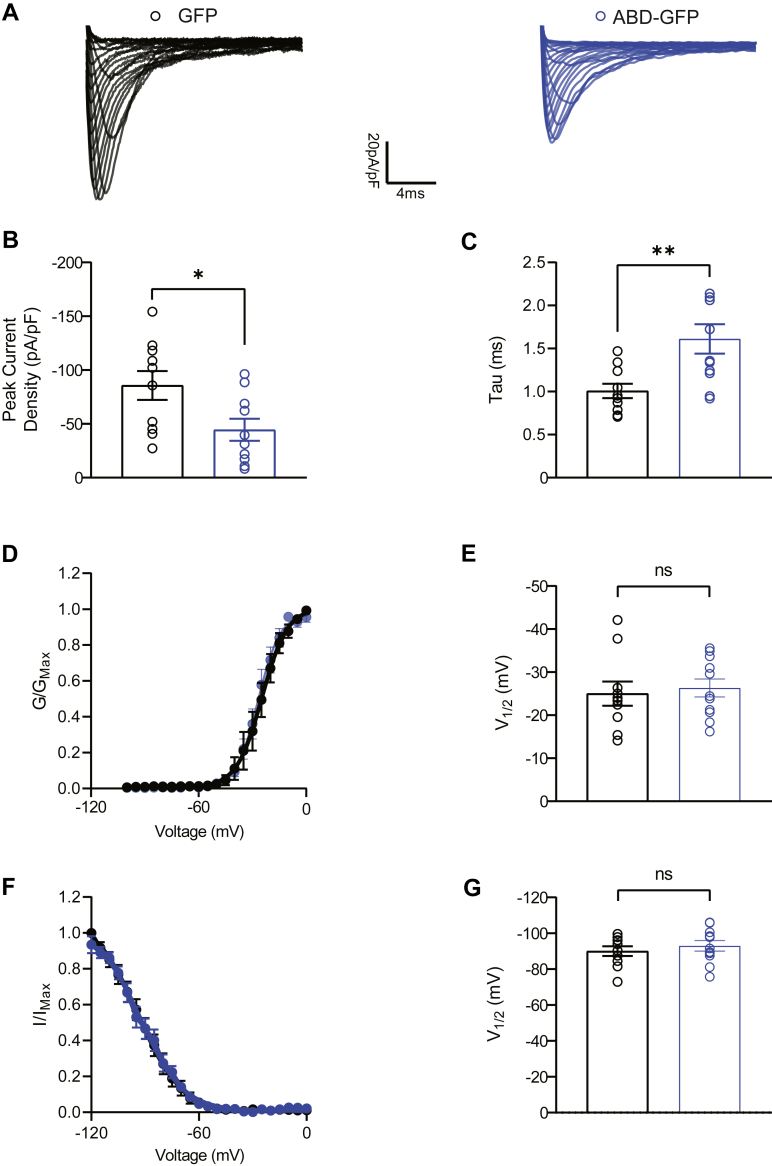
**Functional effects of ABD overexpression on Na**_**v**_**1.6-mediated currents in heterologous cells.***A*, representative traces of transient INa elicited by HEK-Na_v_1.6 cells transfected with GFP (*black*) or ABD-GFP (*blue*). *B* and *C*, comparison of peak I_Na +_ density (*B*) and tau (*C*) between the experimental groups described in (*A*). *D*, normalized conductance plotted as a function of voltage to characterize effects of experimental conditions on the voltage dependence of activation of Na_v_1.6-mediated I_Na+._*E*, bar graph representation of the voltage dependence of activation among indicated experimental groups. *F*, normalized current plotted as a function of voltage to characterize the voltage dependence of steady-state inactivation among experimental groups. *G*, bar graph comparison of the voltage dependence of steady-state inactivation among indicated experimental groups. Data are individual replicates with SEM error bars (n = 8–12 cells/group). Significance was assessed using an unpaired *t* test. ∗*p* < 0.05; ∗∗*p* < 0.01. ABD, axin-binding domain; HEK, human embryonic kidney cell line.

To examine the effects of ABD overexpression on Na_v_1.6-encoded currents, rapidly rising and fast decaying transient Na^+^ currents were evoked using a range of depolarizing voltage stimuli. A significant decrease in peak current density was observed in HEK-Na_v_1.6 cells overexpressing GSK3β′s ABD (Fig. 4, *A* and *B*; 49.6 ± 10.5 pA/pF, n = 11) compared with HEK-Na_v_1.6 cells overexpressing GFP alone (85.7 ± 13.4 pA/pF, n = 10, *p* = 0.0459). This result is congruent with previous investigations evaluating the effects of GSK3β silencing on Na_v_1.6 activity (10); suggesting that the effects on channel activity observed in the present study are resultant of perturbing the GSK3β–Na_v_1.6 PPI complex. Overexpression of the ABD also induced an increase in the tau of fast inactivation of Na_v_1.6 (Fig. 4*C*; 1.161 ± 0.2 ms, n = 11) compared with GFP control (1.01 ± 0.1 ms, n = 10, *p* = 0.0063). Further analyses were conducted to determine the effects of ABD overexpression on the voltage dependence (V_1/2_) of activation of Na_v_1.6 channels. These results indicated no statistically significant effect on the voltage dependence of activation resulting from ABD overexpression (Fig. 4, *D* and *E*; 25.01 ± 2.8 mV, n = 10) compared with the GFP control group (26.31 ± 2.088 mV, n = 11; *p* = 0.7103). To investigate the effects of ABD overexpression on the V_1/2_ of steady-state inactivation of Na_v_1.6 channels, cells were subjected to a standard two-step inactivation protocol consisting of a preconditioning test pulse followed by a variable test pulse. Cells overexpressing the ABD exhibited a V_1/2_ of 92.99 ± 2.9 mV, n = 10 (Fig. 4, *F* and *G*), which was not significantly different from the control group (90.05 ± 2.7 mV, n = 10; *p* = 0.4707).

Given that the initial ABD-GFP construct is expressed as a fusion protein, interference or effects elicited by the GFP tag were a concern. To address these concerns, an additional ABD construct was generated containing a self-cleaving P2A peptide sequence52 (ABD-P2A-GFP) in order to express the ABD and GFP fluorophore independently (Table 1). Overexpression of this construct in HEK-Na_v_1.6 cells resulted in a nearly identical decrease in peak current density (46.71 ± 7.9 pA/pF, n = 10) compared with GFP control (85.71 ± 13.4 pA/pF, n = 10; *p* = 0.0222) as well as a similar slowing in the tau of fast inactivation (1.45 ± 0.19 ms, n = 10) compared with control (1.01 ± 0.08 ms, n = 10; *p* = 0.0476), although this shift was slightly less dramatic than that imparted by the ABD-GFP fusion protein. Unlike the fusion protein, however, cells transfected with ABD-P2A-GFP exhibited a depolarizing shift in the V1/2 of steady-state inactivation of Na_v_1.6 channels (−81.79 ± 1.2 mV n = 10) compared with GFP control (−90.05 ± 2.7 mV, n = 10, *p* = 0.0249). Despite this alteration, the similarity in directionality and amplitude of the effects of the two constructs on peak current density validate that the modulatory effects on Na_v_1.6 activity are not dependent on GFP fusion. The effect of the ABD-P2A-GFP on steady-state inactivation suggests that, if anything, the GFP tag may hinder the full activity of the ABD when fused.

**Table 1:** Summary of the effects of ABD-GFP and ABD-P2A-GFP overexpression on Na_v_1.6 channel activity compared with GFP control

Condition	Peak current density (pA/pF)	Tau of fast inactivation (ms)	V_1/2_ of activation (mV)	V_1/2_ of steady-state inactivation (mV)
GFP	−85.71 ± 13.4 (10)	1.01 ± 0.1 (10)	−25.01 ± 2.8 (10)	−90.05 ± 2.7 (10)
ABD-GFP	−49.60 ± 10.6 (11)∗	1.61 ± 0.2 (11)∗∗	−26.31 ± 2.1 (11)	−92.99 ± 2.9 (11)
ABD-P2A-GFP	−46.71 ± 7.9 (10)∗	1.45 ± 0.2 (10)∗	−24.2 ± 3.0 (10)	−81.79 ± 1.2 (10)∗

Results are indicated as mean ± SEM with the number of individual experiments shown in parentheses. Significance was assessed using an unpaired *t* test to compare HEK-Nav1.6 cells expressing GFP with ABD-GFP or ABD-P2A-GFP.

∗*p* < 0.05; ∗∗*p* < 0.01.

### GSK3**β** ABD facilitates modulation of Na_v_1.6-driven neuronal excitability

Na_v_1.6 has been well established as a principal mediator of action potential firing in excitable cells (50). Previous investigations from our research group have identified GSK3β as a critical regulator of neuronal excitability through its functional modulation of Na_v_1.6 (10). Therefore, having observed that overexpression of GSK3β′s ABD perturbs GSK3β–Na_v_1.6 complex formation, resulting in functional downregulation of Na_v_1.6 activity; we next sought to determine if GSK3β′s ABD mediates its effects on intrinsic neuronal excitability.

**Figure 5:**
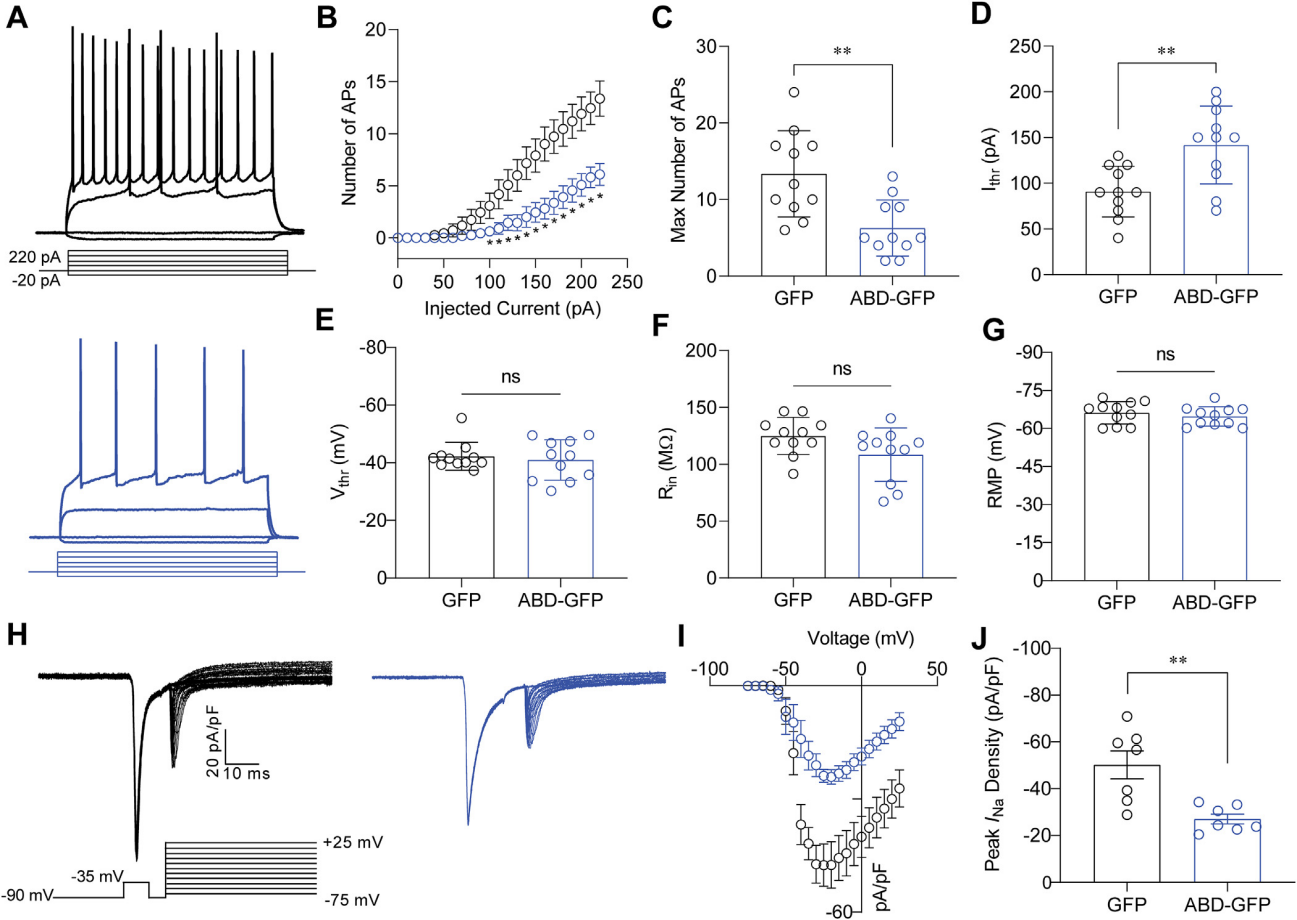
**ABD overexpression reduces excitability and Na**_**v**_**1.6-encoded currents in CA1 pyramidal neurons.***A*, representative traces of evoked action potentials of CA1 cells from mice treated with either GFP (*black*) or ABD-GFP (*blue*) in response to the depicted current-clamp protocol. *B*, number of action potentials fired by CA1 cells of mice treated with GFP (*black*) or ABD-GFP (*blue*) across a range of injected current stimuli (0–220 pA). *C*, maximum number of action potentials evoked from CA1 cells in mice treated with GFP (*black*) or ABD-GFP (*blue*) across a range of injected current stimuli (0–220 pA). *D*–*G*, comparison of current threshold (*D*), voltage threshold (*E*), input resistance (*F*), and resting membrane potential (*G*) between the indicated experimental groups. *H*, representative traces of I_Na+_ elicited by CA1 neurons from mice treated with either GFP (*black*) or ABD-GFP (*blue*) in response to the depicted protocol. *I*, current–voltage relationship of I_Na_+ elicited from CA1 neurons treated with GFP (*black*) or ABD-GFP (*blue*). *J*, Bar-graph comparison of peak current density from the indicated experimental groups. Significance was assessed using an unpaired *t* test. Data shown are individual replicates with SEM error bars (n = 7–8 cells/group). In (*B*), 1 ∗ denotes injected current steps at which *p* is at least <0.05 between the indicated experimental groups. In (*C*, *D*, and *J*) ∗∗ denotes *p* < 0.01. ABD, axin-binding domain.

To test the effects of overexpressing GSK3β′s ABD on intrinsic neuronal excitability, mice were stereotaxically injected in the CA1 region with a vector encoding either GSK3β′s ABD (AAV2-ABD-GFP) or empty vector control (AAV2-GFP). Following a 3-week period to allow for expression of viral particles, acute hippocampal slices were prepared, and whole-cell patch-clamp recordings were obtained to assess the intrinsic excitability of CA1 neurons from the two aforementioned groups. Whole-cell current-clamp recordings were obtained from visually identified CA1 pyramidal neurons, and action potentials were evoked using a range of 800 ms long current injections (Fig. 5*A*). In comparison to CA1 pyramidal neurons expressing the control vector (AAV2-GFP), it was observed that CA1 neurons overexpressing AAV2-ABD-GFP exhibited decreased action potential discharge over the range of injected current stimuli (Fig. 5, *A*–*C* and Table 2). In addition, the current threshold for action potential initiation in neurons overexpressing the ABD was significantly increased (Fig. 5*D* and Table 2; 141.8 ± 12.9 pA, n = 11) in comparison to neurons expressing the control vector (90.9 ± 8.4 pA, n = 11, *p* < 0.005), whereas the voltage threshold for action potential initiation in cells overexpressing the ABD (Fig. 5*E* and Table 2; 40.9 ± 2.1 mV, n = 11) was not significantly different from the GFP control group (42.2 ± 1.5 mV, n = 11 *p* = 0.6232). Finally, regarding passive electrical properties, overexpression of GSK3β′s ABD did not display any significant effects on input resistance (Fig. 5*F* and Table 2) or resting membrane potential (Fig. 5*G* and Table 2) compared with cell expressing of the control vector. Finally, given the effects of ABD overexpression on the intrinsic excitability of CA1 neurons, we next sought to determine if the effects on excitability are resultant of suppressed Na_v_1.6-encoded currents in these cells. To do so, whole-cell voltage-clamp recordings were obtained from CA1 neurons overexpressing GFP or ABD-GFP (Fig. 5*H*). To minimize space-clamp issues, we modified a protocol from Milescu *et al.* (51)*.* that uses a depolarizing prepulse to inactivate Nav channels away from the recording electrode, followed by a second step to record Nav channels near the electrode (52). A significant decrease in peak current density was observed in CA1 neurons overexpressing the ABD (Fig. 5, *I* and *J*) (27.1 ± 2.1 pA/pF, n = 7) compared with GFP control (50.2 ± 5.9, n = 7; *p* = 0.0032). Taken together, these results resemble the effects of GSK3β knockdown on intrinsic excitability and support the hypothesis that overexpression of GSK3β′s ABD prevents native GSK3β from exerting its positive regulatory effects on Na_v_1.6 channel activity and intrinsic excitability.

**Table 2:** Summary of effects of ABD overexpression on intrinsic excitability of CA1 neurons (Fig. 4)

Condition	Maximum number of spikes	Spike I_thr_ (pA)	Spike V_thr_ (mV)	RMP (mV)
AAV2-GFP	13.4 ± 1.7 (11)	90.9 ± 8.4 (11)	−42.2 ± 1.5 (11)	−66.2 ± 1.3 (11)
AA2-ABD-GFP	6.3 ± 1.1 (11)∗∗	141.8 ± 12.9 (11)∗∗	−40.1 ± 2.1 (11)	−64.8 ± 1.2 (11)

Results are indicated as mean ± SEM with the number of individual experiments shown in parentheses. Significance was assessed using an unpaired *t* test to compare cell transfected with ABD-GFP or ABD-P2a-GFP to GFP control.

∗*p* < 0.05; ∗∗*p* < 0.01.

## Discussion

Various investigations implicate GSK3β as an intracellular regulator of ion channels *via* phosphorylation (31, 33), including Na_v_1.6 (10). Notably, however, we previously demonstrated high-affinity binding between GSK3β and the Na_v_1.6 CTD using surface plasmon resonance, a biophysical method used to detect stably formed PPI complexes, indicating that the interaction between the two binding partners is stable and detectable. In addition, it was observed that this interaction occurs in *Escherichia coli*–purified proteins, which notoriously lack post-translational modifications (53). Therefore, we hypothesized that the regulatory interaction between GSK3β and the Na_v_1.6 channel may extend beyond transient phosphorylation and that the full range of GSK3β′s modulatory effects on Na_v_1.6 channel activity are achieved through stable complex formation. In the present study, we show that deletion of GSK3β′s ABD or ABD overexpression perturbs GSK3β–Na_v_1.6 complex assembly, suggesting that this region is necessary for GSK3β–Na_v_1.6 binding, and its overexpression is sufficient for inhibiting GSK3β–Na_v_1.6 complex formation. Investigations of Na_v_1.6 distribution patterns using immunocytochemistry revealed that overexpression of the ABD in HEK Na_v_1.6 cells results in decreased Na_v_1.6 cluster number and density, which is accompanied by a decrease in Na_v_1.6-encoded currents in a manner resembling GSK3β silencing. In CA1 pyramidal neurons, which abundantly express Na_v_1.6 (54) and GSK3β (55), overexpression of the ABD impairs intrinsic neuronal excitability by reducing action potential firing and increasing the action potential current threshold without impacting the resting membrane potential or input resistance, suggesting that the ABD may inhibit regulation of Na_v_1.6 by native GSK3β. Cumulatively, these results provide further evidence for the complex regulatory role of GSK3β on Na_v_ channel activity and implicate the ABD as a critical region in mediating GSK3β′s interactions with Na_v_1.6.

### GSK3**β** ABD is required for complex with Na_v_1.6

In previous investigations from our laboratory, we employed a split-LCA to characterize interactions between Na_v_ channel intracellular CTDs and auxiliary proteins (56). To explore the domains of GSK3β involved in molecular interactions with the Na_v_1.6 CTD, we constructed a series of GSK3β variants with mutations or deletions in regions that might contribute to complex formation with Na_v_1.6.

The Ala mutations and truncation mutants we constructed were guided by the protein's structure and built upon the known functions of GSK3β′s sites and domains. We engineered a single alanine point mutation to serine 9 of GSK3β, a deletion of GSK3β′s activation loop (residues 200–216), a deletion of GSK3β′s ABD (residues 262–299), and a truncation of GSK3β′s C terminus (residues 344–420). Our results indicate no significant effect on GSK3β–Na_v_1.6 complex assembly from mutation to serine 9 of GSK3β or deletion of the activation loop, which suggests that formation of the GSK3β–Na_v_1.6 complex may not be dependent on GSK3β′s enzymatic activity and that GSK3β binds to the Na_v_1.6 CTD *via* a mechanism distinct from its typical coordination of substrates. Truncation of GSK3β′s C terminus (344–420) induced a substantial increase in GSK3β–Na_v_1.6 complex assembly. It has been shown that GSK3β′s C terminus is truncated by calpain I, resulting in increased kinase activity (57, 58). Given the lack of effect resulting from mutation to serine 9, it is unclear whether this increase in binding to Na_v_1.6 related increased kinase function; although deletion of this domain may favor a conformation of GSK3β that more favorably exposes its Na_v_1.6-binding region. GSK3β′s ABD is a unique protein-binding channel composed of an α-helical segment (262–273) and extended loop (285–299). The extended loop (285–299) of this domain displays disordered secondary structure when unbound but is well defined when in complex. This domain represents the interface of GSK3β with multiple binding partners, including Axin, FRAT, and GSKIP (59). Intriguingly, it is observed that the extended loop (285–299) attains divergent conformations depending upon the protein binding partner, and variable combinations of residues within the loop facilitate binding with each partner (36). Here, we show that deletion of GSK3β′s ABD induced a significant reduction in GSK3β–Na_v_1.6 complex assembly, suggesting a critical role for this region in facilitating interactions between GSK3β and the Na_v_1.6 CTD.

### ABD overexpression alters Na_v_1.6 distribution in heterologous cells

If the ABD of GSK3β truly mediates GSK3β–Na_v_1.6 complex assembly, then we hypothesized that expression of this domain alone should be sufficient to perturb binding of full-length GSK3β Na_v_1.6 CTD. Thus, to further validate the importance of the ABD in mediating GSK3β–Na_v_1.6 binding, we generated a construct encoding an ABD-GFP fusion protein to test the effects of ABD overexpression on GSK3β–Na_v_1.6 complex assembly. Overexpression of the ABD, as hypothesized based upon the effect of ABD deletion, induced a decrease in GSK3β–Na_v_1.6 complex assembly compared with GFP control. This finding, combined with the effects of the ABD deletion, suggest that the ABD is not only required for binding of GSK3β to Na_v_1.6 but also that this domain alone is capable of complexing with the Na_v_1.6 CTD.

The contributions Na_v_ channels to excitability are highly dependent on their patterns of expression, subcellular localization, endocytosis, and degradation. In the CNS, clustering of Na_v_ channels is a critical mechanism underlying neuronal conduction. Trafficking of Na_v_ channels to the membrane is a complex and multifaceted process that is regulated *via* post-translational modifications and scaffolding interactions with an array of binding partners. At the neuronal cell membrane, Na_v_ channels are clustered and tethered to the cell membrane in complex with ankyrin G (18, 19, 60), a process that is heavily regulated by phosphorylation. As such, any alterations to the post-translational modifications or protein interactions that facilitate Na_v_ channel trafficking and cell surface expression have substantial impacts on neuronal function.

The intracellular CTD of Na_v_ channels has been identified as a critical motif in Na_v_ channel trafficking (61, 62), and its PPI with this domain mediate Na_v_ channel surface expression and subcellular localization. For example, in the case of Na_v_1.6, we have previously shown that functional interactions between fibroblast growth factor 14 and the CTD of Na_v_1.6 facilitate trafficking to the membrane (63), which is disrupted by disease-related mutations to fibroblast growth factor 14 (20).

GSK3β has been implicated as a regulator of ion channel trafficking (33, 64, 65). Most notably, interactions of GSK3β with the Na_v_1.2 CTD have been shown to regulate surface expression (11), which may confer its effects on Na_v_1.2-encoded currents. Therefore, given the observed perturbation of GSK3β–Na_v_1.6 complex assembly in the presence of overexpressed ABD, we sought to investigate the effects of ABD overexpression on Na_v_1.6 distribution patterns.

Taken with our evidence that the ABD inhibits GSK3β binding to the Na_v_1.6 CTD, overexpression of this domain may impact Na_v_1.6 trafficking *via* various mechanisms. A key contributor to membrane internalization and recycling of Na_v_1.6 is the E3 ubiquitin ligase family neural precursor cell expressed developmentally downregulated protein 4 (NEDD4) (22). NEDD4-2 has been shown to directly interact with the Pro-Ser-Tyr^1945^ recognition motif of the Na_v_1.6 CTD, facilitating channel internalization. Given our findings that pharmacological inhibition of GSK3β perturbs Na_v_1.6 puncta distribution, whereas GSK3β overexpression increases Na_v_1.6 clustering, along with previous investigations indicating that GSK3β inhibition decreases Na_v_1.6 currents (10), it is possible that GSK3β-mediated phosphorylation of T1936 interferes with recruitment of NEDD4-2 to the PxY recognition motif of the Na_v_1.6 CTD, resulting in decreased channel internalization. Overexpression of the ABD may prevent phosphorylation of the Na_v_1.6 CTD by GSK3β, promoting NEDD4-2 recruitment and internalization of the channel, as evidenced by the similar reductions in Na_v_1.6 puncta distribution observed with either GSK3β inhibition or ABD overexpression (66). Another plausible explanation is that the ABD serves as an anchoring point for NEDD4-2. While the phosphorylation state of the ABD in these experiments is unknown, the ABD does contain several proline residues and a potential pT-P motif (GSK3β residues 275–276) and a PxY motif (GSK3β residues 286–288) that may serve as recognition sites for NEDD4 (67, 68), leading to subsequent ubiquitination and internalization of the ABD–Na_v_1.6 complex. While the precise mechanisms *via* which ABD overexpression impacts Na_v_1.6 distribution remains unclear, the observed effect further suggests a role for GSK3β in Na_v_1.6 trafficking that likely contributes to the observed effects on channel activity.

### Functional effects of ABD overexpression on the Na_v_1.6 channel

Whole-cell patch-clamp electrophysiology was employed to observe the effects of ABD overexpression on Na_v_1.6 activity. We have previously shown that GSK3β functionally modulates both Na_v_1.2 (11)- and Na_v_1.6 (10)-encoded currents, which is likely attributed to phosphorylation of their respective CTDs. In the case of Na_v_1.6, we have previously shown that pharmacological inhibition or genetic silencing of GSK3β results in decreased peak current density, whereas GSK3β overexpression elicits an opposite phenotype (10). In the present study, we observed that ABD overexpression in HEK-Na_v_1.6 cells resulted in decreased peak current density in a manner that resembles pharmacological inhibition or genetic silencing of GSK3β. Taken together with previous evidence, this result may suggest that the ABD prevents GSK3β-mediated potentiation of Na_v_1.6 activity by preventing native GSK3β from binding or phosphorylating the Na_v_1.6 CTD.

It has been shown that the effects of GSKβ on Na_v_1.2 and Na_v_1.6 activity are functionally divergent; with GSK3β overexpression potentiating Na_v_1.6-mediated currents (10) but decreasing Na_v_1.2 activity, which is accompanied by a decrease in Na_v_1.2 cell surface expression (11). Therefore, as evidenced by the decrease in Na_v_1.6 puncta observed with ABD overexpression in this study, it is possible that the decrease in Na_v_1.6 peak current density observed here is a result of increased channel internalization; which may also be a result of perturbed GSK3β–Na_v_1.6 complex assembly. Lending further complexity to the effects of the ABD on Na_v_1.6 activity, however, it was observed that ABD overexpression induced slowing of Na_v_1.6 channels into fast inactivation. While the effects of native GSK3β on the tau of Na_v_1.6 fast inactivation have not been fully characterized, these results suggest that the ABD elicits functional effects on the channel resulting from both altered Na_v_1.6 distribution and acute functional kinetics at the plasma membrane, both of which may result from disruption of native GSK3β interacting with the Na_v_1.6 channel. Ultimately, the convergence of effects on Na_v_1.6 peak current density elicited by GSK3β inhibition and ABD overexpression suggests that the ABD may prevent binding of the Na_v_1.6 CTD by native GSK3β, thereby preventing GSK3β-mediated potentiation of Na_v_1.6 activity.

### Effects of ABD overexpression on excitability of CA1 neurons

GSK3β has been thoroughly investigated as a modulator of neuronal excitability (69, 70), and part of this regulation has been attributed to its functional regulation of Na_v_ channels (10, 71). Our previous studies indicate that AAV-mediated genetic silencing of GSK3β results in decreased neuronal excitability, whereas GSK3β knock-in through mutation of its autoinhibitory residues induces an increase (10), implicating GSK3β as a key determinant of intrinsic firing. In this study, we chose to test the effects of ABD overexpression in CA1 pyramidal neurons, as abundantly express GSK3β (55) and Na_v_1.6 is the principal mediator of their excitability (54). We observed that overexpression of the ABD induced a significant decrease in action potential firing and increased the current threshold for action potential initiation, effects that resemble genetic silencing of GSK3β. Action potential initiation in pyramidal neurons is principally regulated by Na_v_1.6 channels, which is reliant upon highly concentrated distribution of Na_v_1.6 channels in the distal AIS (50, 72, 73).

Thus, the observed effects of ABD overexpression on Na_v_1.6 distribution in HEK293 cells (Fig. 2) may predict a similar disruption of Na_v_1.6 trafficking in neurons, resulting in a decreased concentration of Na_v_1.6 channels at the AIS, and thereby diminishing action potential discharge. Moreover, functionally, ABD overexpression resulted in decreased peak current density and slowed the tau of fast inactivation of Na_v_1.6 (Fig. 4 and Table 1), which are both predictive of decreased excitability because of the principal role of Na_v_1.6 in regulating action potential firing (50, 54, 74). Taken together, our finding that ABD overexpression suppressed Na_v_1.6-endoded currents in these neurons, we present strong evidence that overexpression of the ABD decreases excitability *via* modulation of Na_v_1.6.

Our previous studies indicated that either long-term genetic silencing of GSK3β or acute pharmacological inhibition of GSK3β results in decreased neuronal excitability. When the ABD is overexpressed, it is plausible that decreased neuronal excitability is resultant of long-term or acute mechanisms: either perturbed trafficking of Na_v_1.6 to the AIS or a blockade of GSK3β-mediated potentiation of Na_v_1.6 activity at the plasma membrane. Notably, previous study from our laboratory showed that pharmacological inhibition of GSK3β resulted in a significant loss of Na_v_1.6 channels at the AIS of hippocampal neurons (8). Given the observed functional congruence of ABD overexpression and GSK3β pharmacological inhibition, it stands to reason that the ABD induces functional uncoupling of native GSK3β and Na_v_1.6 in neurons, impairing cellular trafficking of Na_v_1.6. In the case of the latter, the ABD may occupy the GSK3β-binding region of the Na_v_1.6 CTD at the plasma membrane, hindering the ability of native GSK3β to phosphorylate Na_v_1.6 at T1936, and thereby decreasing action potential firing through functional downregulation of Na_v_1.6. In either case, the results of the present investigation further a multifaceted role of GSK3β in facilitating neuronal excitability through modulation of Na_v_1.6 distribution and activity.

Overall, using a combination of mutagenesis screening, immunohistochemistry, and electrophysiology, we show that GSK3β′s ABD is a critical mediator of its functional effects on Na_v_1.6 channel activity and neuronal excitability. Given the diverse roles of GSK3β in neuronal activity, neurodevelopment, and disease, our results represent a critical insight into the mechanisms of GSK3β-mediated ion channel regulation.

## Experimental procedures

### Cell culture

All general cell culture reagents were acquired from Invitrogen. HEK293 cells were incubated at 37 ˚C and 5% CO_2_ in a 1:1 mixture of Dulbecco’s modified Eagle's medium and F-12 supplemented with 10% fetal bovine serum, 100 units/ml of penicillin, and 100 μg/ml streptomycin; cells were tested for contamination. The HEK293 cell lines stably expressing the hNa_v_1.6 (12, 40) channels have been previously described and is referred to as the HEK-Na_v_1.6 cell line. The stable HEK293 cell lines were maintained in the presence of additional antibiotics (0.5 mg/ml G418 and 5 μg/ml puromycin) to ensure continuous expression. For transient transfection, cells were transfected at 80 to 90% confluence with equal amounts of plasmid pairs using Lipofectamine 3000 (Invitrogen) according to the manufacturer’s instructions.

### Plasmid preparation

The CD4-Na_v_1.6-NLuc and CLuc-GSK3β constructs were engineered and prepared in the pcDNA3.1 vector as previously described. For GSK3β mutagenesis studies, mutant GSK3β constructs were designed using Geneious Prime software and synthesized by Geneart (ThermoFisher). Mutant CLuc-GSK3β constructs were subsequently subcloned into the pcDNA3.1 vector and sequence verified. The ABD-GFP construct (GSK3β 262–299) was designed using geneious prime software, synthesized by Addgene, and subsequently subcloned into a pAAV-GFP vector (Addgene), and sequence verified prior to use. The ABD-P2A-GFP construct was designed as mentioned previously but incorporated a P2A peptide sequence 5′-GSGATNFSLLKQAGDVEENPGP-3’. The construct was synthesized and cloned into pcDNA3.1 by Addgene.

### Split-LCA

Cells were trypsinized (0.25%), triturated in medium, and seeded in white, clear-bottom CELLSTAR μClear 96-well tissue culture plates (Greiner Bio-One) at ∼0.9 × 10^5^ cells per well in 200 μl of medium. For transiently transfected cells, the trypsinization occurred 48 h post-transfection. The cells were incubated for 24 h, and the growth medium was subsequently replaced with 100 μl of serum-free, phenol red–free Dulbecco’s modified Eagle's medium/F12 medium (Invitrogen) containing inhibitors (0.25–50 μM). The final concentration of DMSO was maintained at 0.3% for all wells. Following 2 h incubation at 37 °C, the reporter reaction was initiated by injection of 100 μl substrate solution containing 1.5 mg/ml of d-luciferin dissolved in PBS (final concentration = 0.75 mg/ml) by the Synergy H4 Multi-Mode Microplate Reader (BioTek). Luminescence readings were performed at 2-min intervals for 20 min, integration time 0.5 s, and the cells were maintained at 37 °C throughout the measurements. Signal intensity for each well was calculated as a mean value of peak luminescence; the calculated values were expressed as percentage of mean signal intensity of the per-plate control samples.

### Immunocytochemistry

HEK-Na_v_1.6 cells transfected with either GFP or ABD-GFP were replated at low density on glass coverslips and then fixed in fresh 4% paraformaldehyde and 4% sucrose in PBS for 15 min. For experiments including treatment with compounds, cells were incubated with vehicle or compound for 1 h immediately prior to fixation. Then, cells were permeabilized with 0.25% Triton X-100 and blocked with 10% bovine serum albumin (BSA) for 30 min at 37 °C. For studies investigating puncta in HEK-Na_v_1.6 cells, cells were incubated overnight at room temperature with a primary rabbit anti-Na_v_1.6 (1:300 dilution, ASC-009; Alomone Labs) diluted in PBS containing 3% BSA. Cells were then washed three times in PBS, incubated for 45 min at 37° C with an anti-rabbit Alexa-647-conjugated secondary antibody (1:200 dilution; Invitrogen), and stained with the nuclear marker 4′,6-diamidino-2-phenylindole (1:1000 dilution; both from Life Technologies) according to the manufacturer’s instructions. For experiments observing expression of CD4-Na_v_1.6-NLuc and CLuc-GSK3β constructs, cells were incubated overnight at 4°C with primary mouse antiluciferase (1:300 dilution, sc-74548; Santa Cruz Biotechnology) and rabbit anti-CD4 (1:300 dilution, ab133313; Abcam) diluted in PBS containing 3% BSA + 0.1% Tween-20. The cells were then washed three times in PBS and incubated with Alexa 488 goat anti-mouse (1:200 dilution, A11001; Invitrogen, ThermoFisher Scientific) and Alexa Fluor 647 goat anti-rabbit (1:200 dilution, A21245; Invitrogen, ThermoFisher Scientific) for 4 h. Coverslips were then washed six times with PBS and mounted on glass slides with Prolong Gold anti-fade reagent (Life Technologies).

### Western blotting

Lysates from transfected cells were prepared, mixed with 4× loading buffer, and heated at 55°C for 10 min. They were then separated on 4 to 20% protein gels (Bio-Rad) at 110 V for 1 h and transferred to a PVDF membrane (Bio-Rad) using 30 V for 2.5 h. The membrane was blocked with 1× Tris-buffered saline 1% Casein Blocker (Bio-Rad) for 1 h on a rocker at room temperature. The membranes were then incubated overnight in a cold room with a blocking buffer containing mouse antiluciferase (1:1000 dilution; Sigma–Aldrich; catalog no.: S8809). The membrane was then washed thrice with a solution of Tris-buffered saline and 0.1% Tween-20, then incubated with horse anti-mouse secondary antibody (1:1000 dilution) conjugated to horseradish peroxidase (Cell Signaling Technology), and visualized using SuperSignal West Femto Maximum Sensitivity Substrate (ThermoFisher Scientific, Pierce). The ChemiDoc Imaging System (Bio-Rad) was used to detect and visualize the proteins.

### Imaging and analysis

Confocal images were acquired using a Zeiss LSM-510 Meta confocal microscope with a 63× oil immersion objective (1.4 numerical aperture). Multitrack acquisition was done with excitation lines at 488 nm for GFP, 543 nm for Alexa 568, and 633 nm for Alexa 647. Respective emission filters were band-pass 505 to 530 nm, band-pass 560 to 615 nm, and low-pass 650. All acquisition parameters, including photomultiplier gain and offset, were kept constant throughout each batch of experiments. Image stacks were analyzed using FIJI (National Institutes of Health [NIH]). Surface-associated puncta were isolated by selecting two optical slices encompassing the top edge from each cell. These two optical slices were then converted to 8 bit grayscale and then merged using maximum projection. Then, cells were segmented using a binary threshold. ImageJ particle analysis was used to detect the puncta in a cell, count their number, and measure their fluorescent intensity value relative to background. This algorithm selected puncta based upon biologically relevant morphological attributes (particle size 0.03–0.5 μm^2^, circularity >0.5). Data were tabulated and analyzed with Microsoft Excel and GraphPad Prism 9 (GraphPad Software, Inc).

### *In vivo* overexpression of GSK3**β** ABD

The pAAV-GFP and pAAV-ABD-GFP plasmids mentioned previously were packaged into AAV2 by the UNC Vector Core. In order to overexpress the ABD *in vivo*, mice were anesthetized with isoflurane (Baxter) and injected bilaterally with control vector (AAV2-GFP or AAV2-ABD-GFP) into the CA1 region of the hippocampus (1 μl/side over 10 min) using stereotaxic coordinates (AP = −2.3, L = 1.7, V = − 1.5). After injection of 1 μl bilaterally, the needles remained in place for 10 additional minutes to allow for spread of the vector and to prevent the vector from spreading up the needle track as previously described (75). Accurate placements were confirmed following brain extraction by visualizing the GFP signal with a Dual Fluorescent Protein Flashlight and VG2 barrier filter glasses (Nightsea). No animals were excluded based upon vector placement. Patch-clamp recordings were performed only from pyramidal neurons expressing GFP in the CA1 region.

### Whole-cell voltage-clamp recordings in heterologous cells

HEK293 cells stably expressing N_a_v1.6 (HEK-Na_v_1.6) were grown in culture as described previously. On the day of recording, cells were dissociated using Gibco TrypLE (Thermo Fisher Scientific) and plated at low density onto glass coverslips situated at the base of wells in CELLSTAR 24-Well Multi-Well Plates (Greiner Bio-One). After allowing cells to adhere for a minimum of 2 h, glass cover slips were transferred to the recording chamber containing 3 ml of extracellular solution comprised of the following salts (millimolar): 140 NaCl, 3 KCl, 1 MgCl_2_, 1 CaCl_2_, 10 Hepes, 10 glucose, pH 7.3. Cells were then incubated in extracellular recording solution containing vehicle (0.1% DMSO) or compound. Recordings were then performed at room temperature (20–22 °C) using a MultiClamp 700B amplifier (Molecular Devices) and borosilicate glass pipettes (resistance of 3–6 MΩ) containing intracellular solution containing the following salts (millimolar): 130 CH_3_O_3_SCs, 1 EGTA, 10 NaCl, 10 Hepes, pH 7.3. Settings on the amplifier were then used to estimate membrane capacitance and series resistance and compensated for electronically by 70 to 80%. Prior to digitization and storage, data were acquired at 20 kHz and filtered at 5 kHz. Clampex 9.2 software (Molecular Devices), interfaced to the electrophysiological equipment using a Digidata 1200 analog–digital interface (Molecular Devices), was used to control all experimental parameters. To assess the relationship of INa and voltage, cells were evoked by depolarizations to test potentials ranging from −100 to +60 mV from a holding potential of −70 mV, followed by a voltage prestep pulse of −120 mV. To assess steady-state inactivation of Nav channels, HEK-Na_v_1.6 cells were evoked using a paired-pulse protocol in which cells were stepped, from the holding potential, to varying test potentials ranging from −120 to +20 mV (prepulse) prior to a test pulse to −20 mV. To assess long-term inactivation, HEK-Na_v_1.6 cells were subjected to four depolarizations at 0 mV for 16 ms separated by three recovery intervals at −90 mV for 40 ms.

#### Voltage-clamp data analysis

INa was normalized to membrane capacitance to determine current density by dividing INa amplitude by membrane capacitance. Current density was then plotted as a function of the holding potential to characterize current–voltage relationships. Tau of inactivation was calculated by fitting the decay phase of currents at −10 mV with a one-term exponential function. To determine the voltage dependence of activation, conductance (GNa) was first calculated using the following equation:

$$G_{\text{Na}}=\frac{I_{Na}}{\left(V_{m}-E_{\text{rev}}\right)}$$

where *I*_*Na*_ is current amplitude at voltage V_m_, and E_rev_ is the Na+ reversal potential. Steady-state activation curves were then generated by plotting normalized GNa as a function of test potential. Plotted data were then fitted with the Boltzmann equation to determine V1/2 of activation values using the following equation:

GNaGNa,max=1+eVa−Emk

where G_Na_, max is the maximum conductance, Va is the membrane potential of half-maximal activation, E_m_ is the membrane voltage, and k is the slope factor. To assess steady-state inactivation, INa normalized to max INa (INa/INa, Max) at the test potential was plotted as a function of prepulse potential. Data were then fitted with the Boltzmann function to determine V1/2 using the following equation:

INaINa,max=11+eVa−Emk

where Vh is the potential of half-maximal inactivation, Em is the membrane voltage, and k is the slope factor. For long-term inactivation, the peak INa of depolarization cycles 2 to 4 was normalized to the peak INa of depolarization cycle 1 (INa/INa, cycle 1) and plotted as a function of depolarization cycle. Data analysis was performed using Clampfit 11.1 (Molecular Devices) and GraphPad Prism 9 software. Results were indicated as mean ± SEM. Statistical significance was determined *via* unpaired *t* tests comparing 0.1% DMSO to an experimental group, with *p* < 0.05 being considered statistically significant.

### Animals

Mice (B6129SF2/J; catalog no.: 101045) were purchased from Jackson Laboratory. Mice were housed in the University of Texas Medical Branch vivarium, which operates in compliance with the US Department of Agriculture Animal Welfare Act, the NIH Guide for the Care and Use of Laboratory Animals, the American Association for Laboratory Animal Science, and Institutional Animal Care and Use Committee approved protocols.

### Acute brain slice preparation

*Ex vivo* electrophysiological recordings were performed in acute hippocampal brain slices. To prepare slices, mice were anesthetized using isoflurane (Baxter) and quickly decapitated. Following decapitation, brains were removed and 300 μM coronal slices containing the hippocampus were prepared using a vibratome (Leica Biosystems) in a chilled and continuously oxygenated (mixture of 95% O_2_/5% CO_2_) Tris-based artificial cerebrospinal fluid (aCSF) composed of the following salts: 72 mM Tris–HCl; 18 mM Tris–base; 1.2 mM NaH_2_PO_4_, 2.5 mM KCl; 20 mM Hepes; 20 mM sucrose; 25 mM NaHCO_3_; 25 mM glucose; 10 mM MgSO_4_, 3 mM sodium pyruvate; 5 mM sodium ascorbate; and 0.5 mM CaCl_2_ (pH = 7.4 and osmolarity = 300–310 mOsm). Prepared slices were then quickly transferred to a continuously oxygenated and 31°C recovery chamber containing fresh Tris-based aCSF for 15 min. Next, slices were transferred to a continuously oxygenated and 31°C chamber containing standard aCSF, which contained following salts: 123.9 mM NaCl; 3.1 mM KCl; 10 mM glucose; 1 mM MgCl_2_; 2 mM CaCl_2_; 24 mM NaHCO_3_; and 1.16 mM NaH_2_PO_4_ (pH = 7.4 and osmolarity = 300–310 mOsm). Slices were then allowed to recover for an additional 30 min prior to recording.

### *Ex vivo* electrophysiology

For the whole-cell patch-clamp recordings performed in current-clamp mode, the prepared brain slices were transferred and submerged in a recording chamber and perfused with standard recording aCSF, which was continuously oxygenated with 95% O_2_ and 5% CO_2_ (pH 7.4). The flow rate in the recording chamber was kept at 1.5 ml/min, and the bath temperature was maintained at 30 to 32 °C by an inline solution heater and temperature controller (TC-344B; Warner Instruments). In addition, 20 μM bicuculline; 20 μM NBQX; and 100 μM AP5 (Tocris) were applied prior to recording to block synaptic activity. Pyramidal neurons were visually identified, and whole-cell current-clamp recordings were performed using an Axopatch 200B or Multiclamp 700B amplifier (Molecular Devices). Borosilicate glass pipettes with a resistance of 3 to 6 MΩ were made using a Narishige PP-83 vertical Micropipette Puller (Narishige International, Inc). Current-clamp recordings were performed with pipettes filled with internal solution containing (in millimolar): 145 potassium gluconate, 2 MgCl_2_, 0.1 EGTA, 2 Na_2_ATP, and 10 Hepes (pH 7.2 with KOH; and osmolarity = 290 mOsm). Prior to seal formation, the pipette potential was adjusted to zero, and the pipette capacitance was compensated. Membrane potentials were not corrected for the liquid junction potential.

*Ex vivo* recordings performed in voltage-clamp mode were performed similarly to current-clamp recordings acquired from CA1 neurons, with a few differences. After incubation in compound or vehicle, slices were transferred to a recording chamber perfused with continuously oxygenated and heated standard aCSF (however, for voltage-clamp recordings, the aCSF was supplemented with 120 μM CdCl_2_ to block calcium channels). Voltage-clamp recordings of medium spiny nerurons were performed using pipettes filled with the following intracellular solution: 100 mM cesium-gluconate (Hello Bio, Inc); 10 mM tetraethylammonium chloride; 5 mM 4-aminopyridine (4-AP); 10 mM EGTA; 1 mM CaCl_2_; 10 mM Hepes; 4 mM Mg–ATP; 0.3 mM Na3–GTP; 4 mM Na2–phosphocreatine; and 4 mM NaCl (pH = 7.4 and osmolarity = 285 ± 5 mOsm/L; CsOH used to adjust pH and osmolarity; all salts except cesium-gluconate were purchased from Sigma–Aldrich). After GΩ seal formation and entry into the whole-cell configuration, a cocktail of synaptic blockers (20 μM bicuculline; 20 μM NBQX; and 100 μM AP5 [synaptic blockers purchased from Tocris]) was perfused to block synaptic currents along with 1 μM ICA12142 and 10 nM Phrixotoxin3 to block currents mediated by Na_v_1.1 channels and Na_v_1.2 channels, respectively. The voltage-clamp protocol shown in Figure 5 was then performed as previously described (42), and acquired data were analyzed as previously described (16, 42).

#### Current-clamp data analysis

In current-clamp recordings, the maximum number of APs was determined by quantifying the maximum number of APs a CA1 pyramidal cell fired at any current step during the evoked protocol (current injections ranged from −20 to +210 pA, 800 ms). The average instantaneous firing frequency was determined by calculating the mean value of the instantaneous firing frequency between APs at a given current step. The current threshold (Ithr) was defined as the current step at which at least one AP with overshoot was evoked. The voltage threshold (Vthr) was defined as the voltage at which the first-order derivative of the rising phase of the AP exceeded 10 mV/ms (from a plot of dV/dt *versus* V). Passive membrane properties, such as input resistance (Rin) and membrane time constant (τ), were measured with current-clamp recordings with a membrane potential of −70 mV. To determine Rin, the steady-state values of the voltage responses to a series of current steps from −120 to +20 pA with 20 pA increments/step and a duration of 500 ms were plotted as a voltage–current relationship. Rin was calculated as the slope of the data points fitted with linear regression. Membrane τ was calculated by fitting a single exponential function to the first 100 to 150 ms at a −40-pA hyperpolarizing, 500 ms current step from the same series. Cm was estimated as tau/Rin × gain of the amplifier (usually 1000). Data analysis was performed using pClamp 9 (Clampfit 9) (Molecular Devices) and Origin 8.6 or Prism, version 9.1.0, and the results were plotted with Origin 8.6 (OriginLab Corp) or Prism, version 9.1.0 (GraphPad Software, Inc).

## Data availability

The data used and analyzed in this study, as well as any source data supporting the present work, are available from the corresponding author upon reasonable request.

## Supporting information

This article contains supporting information.

## Conflict of interest

The authors declare that they have no conflicts of interest with the contents of this article.
