# Supplementary material for: Axin-binding domain of glycogen synthase kinase 3β facilitates functional interactions with voltage-gated Na+ channel Nav1.6
Source: J Biol Chem. 2025 Jan 8;301(2):108162. doi: 10.1016/j.jbc.2025.108162 (PMC11847078; doi:10.1016/j.jbc.2025.108162)
Supplement: Supporting Information [file mmc1.docx]

**Axin-binding domain of glycogen synthase kinase 3β facilitates functional interactions with voltage-gated Na+ channel Nav1.6**

Baumgartner, T.J.; Dvorak, N.M.; Goode, N.A.; Haghighijoo, Z; Marosi, M.; Singh, A.K.; Singh, J.; Laezza, F

Department of Pharmacology and Toxicology, University of Texas Medical Branch, Galveston, TX 77555

Corresponding Author: Fernanda Laezza feleazza@utmb.edu

**Supporting Information**

Contents:

Figure S1. Expression of Luciferase Constructs and Nav1.6 Intensity Plot

**Figure S1 – Expression of Luciferase Constructs and Nav1.6 Intensity Plot**


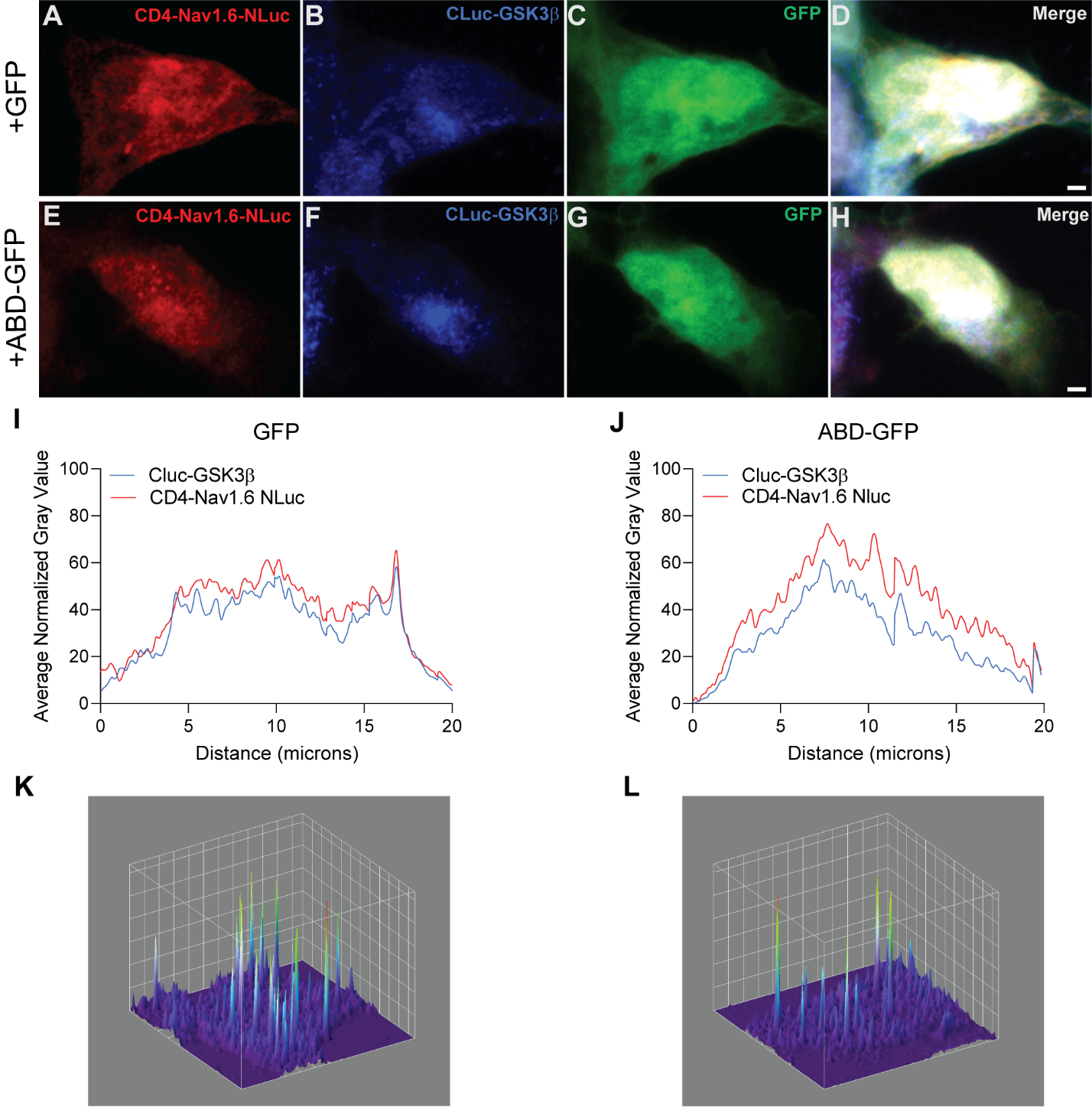
 Figure s1. (A-H) Representative Airyscan images showing CD4-Nav1.6-NLuc immunofluorescence (Red) along with CLuc-GSK3β (blue) in HEK293 cells transiently transfected with GFP (A-D) or ABD GFP(E-H). Representative Airyscan images show CD4-Nav1.6-NLuc (A,E; red); CLuc-GSK3β(B,F, blue); GFP(C, green); ABD-GFP(G, green); or merged channels (D,H) in transiently transfected HEK293 cells. (I, J)Average intensity profiles of CD4-Nav1.6-NLuc and CLuc-GSK3β immunofluorescence derived from region of interest (ROI) drawn over *n* = 7-11 cells transfected with either GFP (I) or ABD-GFP(J). Note that the intensity profiles of the green and blue covary along the ROI. (K,L) Intensity plots of Nav1.6 immunofluorescence generated from cells transfected with either GFP (K, main Figure 2D-H) or ABD-GFP (L, main Figure 2I-M).
